# Expectations and requests regarding team training interventions to promote interdisciplinary collaboration in medical rehabilitation – A qualitative study

**DOI:** 10.1186/s12909-015-0413-3

**Published:** 2015-08-19

**Authors:** C. Müller, A. Plewnia, S. Becker, M. Rundel, L. Zimmermann, M. Körner

**Affiliations:** 1 Medical Psychology and Medical Sociology, Medical Faculty, University of Freiburg, Hebelstr. 29, 79104 Freiburg, Germany; 2 Saarland University of Cooperative Education in Health Care and Welfare, Saarland, Germany; 3 Celenus Kliniken Offenburg, Department of Quality Management, Offenburg, Germany

## Abstract

**Background:**

Interdisciplinary teamwork and team interventions are highly valued in the rehabilitation sector because they can improve outcomes of care for persons with complex health problems. However, little is known about expectations and requests regarding team interventions, especially in medical rehabilitation. This study aimed to explore how clinical managers and health professionals within multidisciplinary rehabilitation teams describe their expectations and requests regarding team-training interventions in the field of medical rehabilitation.

**Methods:**

Considering the methodology of qualitative research, data were collected using semi-structured interviews and focus groups at five rehabilitation clinics in Germany. We conducted face-to-face interviews with 5 clinical managers and 13 department heads of health care teams as well as five focus groups with a total of 35 members of interdisciplinary rehabilitation teams. Afterwards, the data were analyzed through qualitative content analysis encompassing data coding and using inductive thematic analysis.

**Results:**

The exploration of team members´ and clinical managers´ descriptions showed that, to them, interdisciplinary team training programs should include a wide array of training contents. Seven common core themes emerged from the interviews, including participation of employees, leadership, communication, team meetings, team composition, coordination, and equal esteem. Additionally, 13 themes were identified by either managers or team members. The body of expectations regarding team training content in healthcare spans the continuum of changes on the team and organizational levels. On the organizational level, a number of structural factors were mentioned (e.g. improving the general conditions for team meetings, organized workshops to exchange interdisciplinary experiences, and leadership training), and on the team level, changes in procedural factors were listed (e.g. optimizing the consecutive planning and coordination of patient treatments, clarity with regard to roles and responsibilities of team members, and mutual esteem and appreciation between different professions).

**Conclusions:**

The synthesis underscores that there is meaningful heterogeneity in team training needs; training interventions should be locally adapted for each clinic in terms of training content and training strategies. Tailored team interventions are important for rehabilitation clinics. Future work should evaluate employed team training concepts over time as well as training contents, implementation strategies, and learning outcomes. This includes using robust study designs and evaluating team-training effects.

## Background

The complex health problems of patients in rehabilitation settings and the changing demands in health care delivery (e.g. altered disease spectrum, medical developments in treating patients, and changing needs of patients) require effective and efficient cooperation between the various health care professionals as well as coordination among the entire health care team [1, 2]. An interdisciplinary health care team is a group of colleagues from two or more disciplines who coordinate their expertise in providing care to patients. Ideally, they meet regularly in collegial discussion about each patient, exchange information, analyze the patient’s problems, develop a treatment plan, and cooperate to fulfill the team tasks [3]. To tackle these complex dynamic and sometimes difficult tasks, interdisciplinary teamwork skills are needed, which are competencies that individual team members must possess in order to perform teamwork and treat patients [4, 5]. In this context, interdisciplinary teamwork and team training interventions are highly valued in the rehabilitation sector because they are thought to improve outcomes of care [6–9]. But in practice, interdisciplinary teams do not always work well [3]. Although healthcare professionals are often required to work in team environments, most have not had sufficient opportunities to learn with, from, and about other healthcare professionals [2]. However, Baker et al. 2005 [6] pointed out that members of health care teams are rarely trained together in rehabilitation settings and often come from different disciplines and diverse educational programs.

In previous reviews, Buljac-Samardzic et al. [8], Rabøl et al. [10], Eppich et al. [11], Dietz et al. [12], and Weaver et al. [13] stated that team training has been mostly implemented across a broad range of acute care contexts, especially in high-risk environments (e.g. obstetric care, emergency care, or critical care). This includes academic hospitals, community-based hospitals as well as medical centers affiliated with the US Department of Veterans Affairs (VA), but team training has rarely been done in rehabilitation or long-term care settings. The need to train health professionals who can work across disciplines is seen as essential for effective health care delivery, especially in view of the expected growing importance of rehabilitation. Team training interventions have been shown to improve team outcomes (learner knowledge, self-efficacy, teamwork attitudes, teamwork climate, or safety climate), clinical care processes, quality assurance, and even patient outcomes [2, 7, 8, 14, 15]. Team training can serve to ensure that teams have a shared understanding of their purposes, goals, and the behaviors necessary to work effectively by generating shared knowledge among team members. It also facilitates shared decision-making and coordination and enables continuous team self-correction [5]. In general, team training is an overarching term that encompasses a broad range of learning and development strategies, methods, and teamwork skills [13]. Team training activities are often designed to develop generalizable, portable teamwork skills that learners can apply across different settings and health care teams. They are characterized by a constellation of content (e.g. knowledge, skills, and attitudes), tools (e.g. checklists, communication tools, and performance measures), and training methods (information, demonstration, and practice-based learning methods). In this sense, team training is a methodology for optimizing the communication, coordination, and collaboration of healthcare teams by combining specific content, methods, and tools to support the transfer of training into daily practice as well as to provide post-training support through coaching and ongoing measurement (teamwork process evaluation or feedback) [13, 16].

Overall, team training programs in healthcare vary in the training modalities and methods used. Training methods can be conceptualized in terms of three broad categories: (1) information-based methods (e.g., didactic lecture), (2) demonstration-based methods (e.g., behavioral modelling and videos), and (3) practice-based methods (e.g., simulation and role-playing). The literature review by Weaver et al. 2010 [7] found that the majority (83 %) of team training programs integrated both information-based and practice-based methods, while only 35 % reported incorporating demonstration-based activities. In terms of implementation strategy, both train-the-trainer and direct train-the-staff strategies have been used. It is known that such team training programs tend to be discipline-specific and even organization-specific and that the straightforward transfer of training from one work setting to another has often been ineffective and problematic, particularly in different healthcare systems [17].

No evidence-based team intervention programs are currently available for the German medical rehabilitation context; therefore, we intend to conceptualize a team-training program for this setting. But how can we develop team-training programs or develop these teams? How do we know which actual team needs or purposes are important or relevant for healthcare professionals in medical rehabilitation clinics? This qualitative study was motivated by these questions, and it provides a picture of research conducted on team training in terms of healthcare team members´ expectations and concerns. This study aimed to explore how clinical managers and health professionals within a multidisciplinary rehabilitation team describe (a) their common expectations and requests regarding team training (Study Aim 1) and (b) their diverging expectations and requests regarding team training interventions (Study Aim 2) in medical rehabilitation. We wanted to answer the following research question: What are clinical managers’, department heads’, and health professionals´ expectations and requests regarding the development of team training interventions?

## Methods

### Study design and setting

A qualitative research approach was used in order to explore the expectations and concerns regarding team intervention programs of clinical managers, department heads of rehabilitation teams, and health care professionals. We conducted semi-structured interviews [18, 19] and focus groups [20–22] guided by theoretical concepts of a health-related team model [23, 24] as well as the model of integrated patient-centeredness [25] in order to triangulate the findings from a team leader perspective on the one hand (clinical managers/department heads) and a team member perspective on the other hand. The epistemological framework of our study is a systems theory approach that recognizes that organizations have boundaries and that transactions occur within the system and its sub-systems as well as in the wider context of organizations and dynamics of the environment [26]. Systems theory allows us to understand the behavior of systems and sub-systems and offers a dynamic view of teamwork. From a systems theory point of view, individual team members cannot be understood in isolation from one another, and a team cannot be fully understood without understanding the organizational context within which it exists. We used two methods of data collection. On the one hand, we chose face-to-face interviews with clinical managers and department heads because our research objective was to explore and understand the individual views of managers about expectations and requests regarding team training interventions without influence of team members´ points of view. In addition, we suspected that in the presence of supervisors, team members would not be open and might express themselves cautiously about the topic because clinical managers are still viewed as persons worthy of respect. With the focus group method, on the other hand, we included an excellent approach for generating ideas focused on the particular topic and for collecting data from multiple team members simultaneously. In addition, we assumed that the sense of belonging to a group can increase the participants’ sense of cohesiveness and help them feel safe to discuss and share information without the presence of their supervisors and that in this setting, participants can perhaps suggest possible solutions. This research is part of the umbrella project “Design and evaluation of a patient-centered team training program for medical rehabilitation” funded by the German Federal Ministry of Research and Education and the German Statutory Pension Insurance Scheme (grant number: 01GX1024). In Germany, medical rehabilitation is a branch of rehabilitation medicine that aims to enhance and restore functional ability and quality of life for patients with physical, cognitive, mental, communicative, behavioral, or social diseases, impairments, or disabilities. The emphasis is not on the full restoration to the premorbid level of function but rather the optimization of quality of life for those not able to achieve full restoration. A wide range of health care professionals, such as rehabilitation-trained nurses, physical therapists, occupational therapists, speech therapists, psychologists, and social workers, are involved in rehabilitation treatment. Multidisciplinary teams provide expertise in the diagnosis, assessment, and multidimensional treatment of patients with disabling disorders, and they work across the range of healthcare settings, in post-acute early rehabilitation clinics, in-patient rehabilitation clinics, and outpatient rehabilitation centers. In this investigation, five large inpatient rehabilitation clinics from different geographic areas in Southwest Germany served as study sites. These clinics differed in specializations, namely cardiology (1 clinic), neurology (1 clinic), orthopedics (3 clinics), and oncology (1 clinic), as well as in size (90 to 210 beds). Data were collected from March to May 2012. According to the recommendations of the EQUATOR network [27], an international initiative for the dissemination of "Guidelines for Reporting of Health Research", the COREQ criteria (Consolidated Criteria for Reporting Qualitative Research) were used in this study [28].

### Recruitment and sampling

We contacted clinical managers individually by letter, informed them about the team-training project, and asked them to participate. The participants recruited were clinical managers, department heads of health care teams, and members of health care teams. To ensure diversity of participants, purposive sampling was used to identify potential health professionals, department heads, and clinical managers. The subjects differed in age, education levels, work experience, and organizational roles. The teams were composed of health care professionals working at the medical rehabilitation clinics and treating patients, such as physicians, psychologists, psychotherapists, physical therapists, nurses, dieticians, occupational therapists, social workers, and speech therapists. To be included in the study, the participants had to (1) have been a member of the rehabilitation team for at least one year, (2) participate in interdisciplinary clinical practice, (3) be 18 years of age or older, (4) speak fluent German and, (5) be willing to participate.

### Face-to-face interviews and focus groups

We conducted a total of 18 face-to-face interviews with clinical managers and department heads of health care teams and five focus groups (interdisciplinary treatment team) with a total of 35 participants in groups of six to ten individuals. All interviews and focus groups were audiotaped and subsequently transcribed. Additionally the focus group discussions were videotaped. The additional recording of focus group sessions via video was necessary to identify the speakers for data transcription. We only used the video tapes to identify the speakers and not for analyzing the data. The focus groups yielded a total of 165 transcript pages (362 minutes total), and the transcripts of the interviews (630 minutes total) covered 214 pages. Participant characteristics are reported in Table 1.

**Table 1:** Sociodemographic information of focus groups (rehabilitation team) & interviews (clinical managers)

Interview Setting	Field	Number of participants (n)	Sex	Age: M, SD, range	Professions
Focus Groups	Oncology	n = 7	female (n = 6)	M = 46.9	physicians (n = 1)
					nurses (n = 2)
				SD = 6.1	psychologists (n = 1)
			male (n = 1)	Range = 36-54	therapists (n = 3)
	Neurology	n = 6	female (n = 5)	M = 42	physicians (n = 1)
					nurses (n = 2)
				SD = 7.6	psychologists (n = 1)
					therapists (n = 1)
				range = 32-51	social workers (n = 1)
			male (n = 1)		
	Orthopedics	n = 6	female (n = 6)	M = 47	nurses (n = 1)
					psychologists (n = 1)
				SD = 9.7	
					therapists (n = 4)
				range = 31-55	
	Orthopedics	n = 6	female (n = 3)	M = 42	physicians (n = 2)
					psychologists (n = 1)
					therapists (n = 2)
				SD = 11.5	
					social workers (n = 1)
			male (n = 3)		
				range = 28-58	
	Orthopedics	n = 10	female (n = 2)	M = 45.3	physicians (n = 2)
					nurses (n = 2)
					therapists (n = 5)
				SD = 11.4	social workers (n = 1)
			male (n = 8)		
				range = 23-59	
Interviews	Oncology	n = 4	female (n = 2)	M = 52.5	department head psychology (n = 1)
					chief physician (n = 1)
					department head physical therapy (n = 1)
				SD = 6.3	
			male (n = 2)		nursing manager (n = 1)
				range = 49-62	
	Neurology	n = 5	female (n = 1)	M = 48.0	department head psychology (n = 1)
					chief physician (n = 1)
					department head physical therapy (n = 1)
				SD = 5.5	department head speech therapy (n = 1)
			male (n = 4)		
				range = 40-54	department head occupational therapy (n = 1)
	Orthopedics	n = 4	female (n = 2)	M = 51.0	department head psychology (n = 1)
					chief physicians (n = 1)
				SD = 4.5	department head physical therapy (n = 1)
			male (n =2)		
					nursing manager (n = 1)
				range = 46-57	
	Orthopedics	n = 3	female (n = 0)	M = 45.0	chief physician (n = 1)
					department head physical therapy (n = 1)
				SD = 7.8	
				range = 36-50	nursing manager (n = 1)
			male (n = 3)		
	Cardiology	n = 2	female (n = 1)	M = 48.5	chief physician (n = 1)
				SD = 0.7	
					department manager physical therapy (n = 1)
				Range = 48-59	
			male (n = 1)		

Seven interviews were conducted in the field of orthopedics, five in neurology, four in oncology, and two in cardiology. The sample covered the typical hierarchical levels and functional sections of rehabilitation clinics. Interviewees included five chief physicians, three nursing managers, seven therapeutic department heads, and three department heads of psychology departments. The interview participants had a mean age of 49.2 years (SD = 5.7). The age of the participants ranged between 36 to 62 years, and the majority of participants were male (N = 12; 66 %). The range of interview duration was between 28 and 47 minutes. One focus group discussion of the interdisciplinary treatment team was conducted at each rehabilitation clinic. In total, 35 members of the health care team participated, including six physicians, seven nurses, fifteen therapists (speech therapists, occupational therapists, and physical therapists), four psychologists, and three social workers. Twenty-two participants were female (63 %) and thirteen were male (37 %). The average age was 44.7 years (SD = 9.33), with a range of 23 to 59 years (see Table 1).

### Procedure and ethical considerations

The interviewers, a psychologist (LZ) and an occupational therapist (CM), informed the participants about the study. The participants received a written information sheet on the study prior to providing consent. At the beginning of the interview, the purpose of the research, interview method, procedures, risks and benefits of the study, and data privacy and confidentiality were explained to the participants. The subjects were informed that they had the right to withdraw from the study at any time. All participants indicated their willingness to participate by signing the consent form. The study was conducted in accordance with the German Federal Data Protection Act (BDSG in the version released June 11, 2010) regarding data collection, storage, processing, and analysis [29]. To ensure the confidentiality of the information, all participants were assigned a pseudonym code number, and the clinics were similarly assigned pseudonyms. Research participants were informed regarding compliance with the data protection law, anonymity, and the principles of free consent and voluntary participation in the study. The recorded interviews and focus groups were transcribed by experienced independent transcribers who signed written confidentiality agreements. The study was performed in accordance with the ethical principles for medical research of the World Medical Association Declaration of Helsinki [30]. The Ethics Committee of the University of Freiburg approved the study (official approval number: 190/12).

### Data collection

In accordance with qualitative research methodology [31], the interviews and focus groups were conducted by two researchers (CM, LZ) from the project group. Each face-to-face interview was conducted by one of the researchers (CM, LZ), while focus groups were held by both of them collaboratively. One of the researchers guided the focus groups, while the other researcher took field notes. The combined use of distinct data collection strategies made it possible to acquire rich material for analysis. While the interview method allowed us access to information that participants may not want to share publicly, the focus groups made it possible to obtain opinions on socially debated topics [32].

The semi-structured qualitative interviews and focus groups were supported by an interview guide [33, 34]. This interview guide, which included predetermined topics, was developed to explore the health professionals’ perspectives, requests, and expectations regarding team interventions. It was used as a checklist to guarantee that all topics were discussed and served as a theoretical framework for the current interview study. This guide was developed based on theoretical concepts of a health-related team model [23, 24], the model of integrated patient-centeredness [25, 26] as well as discussions within the research team and with experts in the field of medical rehabilitation. The questions had an open-ended format to bring up topics while giving respondents the opportunity to talk freely about their experiences and perspectives in their own words. During each interview, specific questions were asked, for example, “Please talk about your experiences with teamwork”, “What problems do you face during interdisciplinary practice?”, and “What do you expect from team intervention programs?” Additional questions were asked when necessary, for instance, “Could you elaborate more on your experience?”. The interview guide was pilot tested in collaboration with a scientist experienced in qualitative methods (MK). After finishing the interviews, a brief questionnaire on socio-demographic information and professional practice was completed with the participants.

### Data analysis

To analyze the data, an inductive thematic analysis approach as described by Mayring [35, 36] was used in order to identify themes across the data that are relevant to the research question. The transcription of the interviews and focus groups was carried out using the transcription software F4 [Audiotranskription, Dr. Dresing & Pehl GmbH] and transcription rules by Dresing & Pehl [37]. During the data preparation phase, each transcript was read by three authors (CM, AP, LZ) to gain an understanding of the whole content and to ensure validity of the transcripts [38]. The transcripts were reread systematically line-by-line by two of the authors (CM, AP) to identify and underline the meaning units of text that were relevant to the research aim. All interview transcripts were imported into the qualitative analysis software program MAXQDA® 10 (VERBI software GmBH, Germany) to facilitate data organization and analyses for coding and retrieval. During the open coding phase, two researchers (CM, AP) independently coded the interviews. The meaning units were condensed, abstracted, and labeled with codes. Further, these codes were used to develop main categories. The codes were compared to each other to distinguish similarities and differences related to the research question. Main categories were formed from the interviews until saturation was achieved and no new information could be revealed [31, 39, 40]. To increase credibility and trustworthiness, several steps were taken to verify the results. The researchers met over several meetings to discuss the developing analysis. This step allowed for enhanced reflexivity and ensured rigor. A third researcher (MK) independently reviewed the codes to ensure consistent interpretation with identified themes and to verify that themes were adequately summarized.

## Results

**Fig. 1:**
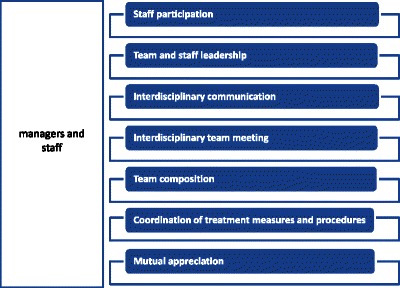
Common concerns of managers and staff

**Fig. 2:**
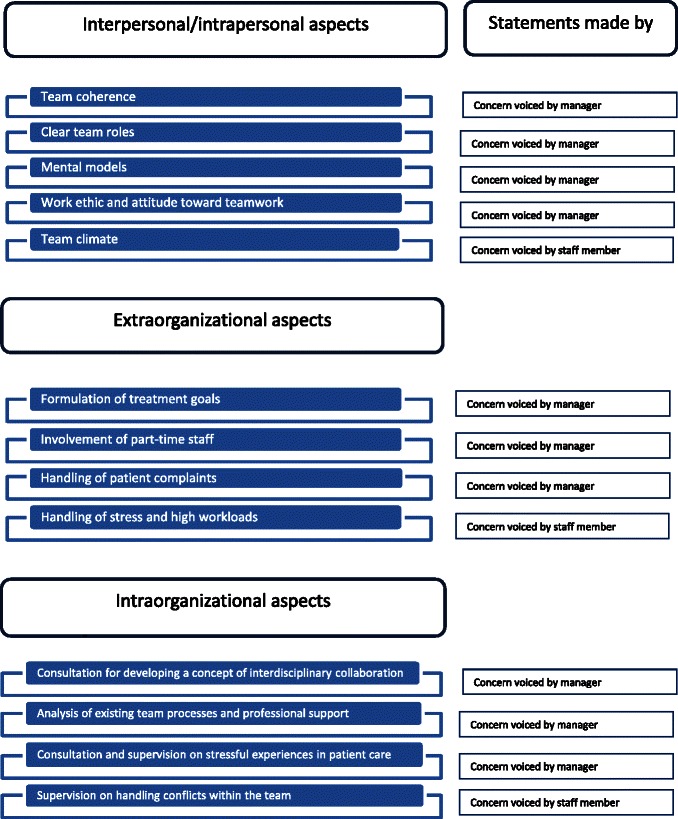
Expectations of team training interventions without overlap between managers and rehabilitation teams

The team development expectations expressed by managers and members of the rehabilitation teams are shown for all centers combined in the form of structured summaries (Results Part A) and in table form (Results Part B) [41, 42]. The summaries are based on the original statements from the empirical data, described in paraphrased form with typical examples. First, the common responses of managers and members of the rehabilitation team were compared and contrasted in terms of content and topics (Study Aim 1, see Fig. 1), and in a second step, differences between the two groups were discussed (Study Aim 2, see Fig. 2). The described differences in expectations are related to aspects that were listed either only by managers or only by team members.

### Part A: Common expectations regarding team training interventions (Study Aim 1)

We created seven categories (see Fig. 1) that reflect different facets of the common expectations regarding team development held by both managers and members of the rehabilitation team. These expectations are related to optimizing interdisciplinary collaboration and address contents of team training interventions that are depicted in Fig. 1 and are described below in the form of thematic summaries. The following data excerpts, which represent statements of the respondents, were labelled as follows: “C” for “Clinic”,”I” for”Interview”, “F” for “Focus group”, and “L” for “Line”. Of the 79 thematic expectations, 48 were expressed by managers and 31 by the rehabilitation teams.

#### Staff participation

Both managers and team members were concerned about staff participation. In this regard, managers particularly focused on involving staff members with the following goals: (a) achieving better involvement of part-time staff in daily routine on the ward, (b) strengthening staff motivation, (c) optimizing the integration of knowledge of the various professions, (d) improving attention to the interests and concerns of individuals, (e) promoting communication between department heads and members of the rehabilitation team, and (f) involving staff members in organizing work hours. As regards the knowledge integration stressed by managers, they considered the coordinated inclusion of the expertise of all professional groups as relevant since knowledge integration contributes to the development of interdisciplinary treatment planning and execution. They emphasized that familiarity with the concepts and focus areas of related professions is essential for everyday cooperation. Furthermore, managers highlighted the idea that performance appraisal interviews can strengthen motivation: *"I also strive to maintain staff motivation this way, one should probably say, or to improve it and incorporate ideas. They often have better solutions as well. They often are the experts; sometimes I am far removed from certain topics. […] It is important to me, it is not always successfully done, that all interests are taken into account in this area. But I think that is the right idea, to involve someone"* [C1_I.2_L.178-180]. Members of the interdisciplinary treatment teams were also concerned with greater involvement in decision-making processes as regards (a) the handling of complaint management, (b) information and communication management, (c) the implementation of treatment concepts, and (d) the nature of communication processes within the team. To improve the conditions for interdisciplinary communication processes within the team, they wanted greater consideration and direct incorporation of their ideas. Team members stated it would be desirable to have their experience-based knowledge considered and their voice heard in case of changes in treatment processes or in the implementation of new treatment concepts and wanted greater participative involvement in this regard.

#### Team and staff leadership

Both groups saw a need for training on team and staff leadership. Managers and the members of the rehabilitation team alike considered this area relevant, albeit with different goals. Managers were particularly concerned with manager training. They argued that team and staff leadership training should be established on the department head level to achieve greater staff satisfaction and to better prepare members of middle management for fulfilling their responsibilities. Furthermore, managers suggested conducting workshops inviting leaders from various regional rehabilitation facilities in order to facilitate experience exchange and to help them learn from one another. In this context, managers considered team training enriching and important. Yet, 16 of 18 interviewed managers stated that they had not offered any specific courses or team training on team or staff leadership or on interdisciplinary collaboration in recent years. At some rehabilitation facilities, discipline-specific and interdisciplinary case discussions took place, but they did not focus on team leadership aspects. One manager noted: *"Training, well, there is continued education, which takes place regularly since last year, and center-related topics are discussed. It's about the orthopedic treatment pathway, the cardiological treatment pathway (…). Yes. But training specifically about teamwork, no, I can't think of any right now …"* [C4_I.16_L.38-42]. Like the managers themselves, the rehabilitation teams stated that there is a need for manager training. From the perspective of the rehabilitation teams, leadership skills to be covered should include social skills and the appreciative, respectful interaction with staff as well as the methodological skill of giving appropriate feedback. Furthermore, the rehabilitation teams believed that department heads and physicians require communication training on interacting respectfully in management-to-staff communication.

#### Interdisciplinary communication

Both managers and the members of the rehabilitation team listed communication as a concern in team development. They desired improvements in transparency and information flow (a) within rehabilitation teams, (b) within department subteams, and (c) in the overall organization. Furthermore, both team members and the management wanted to focus on the communication between treatment providers and patients as well as on respectful communication with one another.

This reflects both verbal and nonverbal communication with sensitivity, clarity, and self-awareness of opinions, beliefs, and perspectives within the team and between team members. One team member of the focus group stated: *"(…) well, I think that when clinical managers make decisions that do affect the team, the occupational groups should be involved if it's about improving communication at the interfaces. For the communication between the individual departments to be more intensive. And (…) I would just like for us to practice more open and respectful communication … I think that greatly influences our well-being and satisfaction in the team … I hope training can help with that."* [C4_F.4_L.523-428]. Managers expressed a need for fixed time windows and sufficient time for interdisciplinary and intradisciplinary communication between members of the rehabilitation team, between heads of department, and between center management and department heads. With team training, they associated the opportunity to initiate change processes and potentially influence the conditions under which teamwork takes place. Managers hoped to benefit from team training by collecting suggestions and ideas about optimizing communication and about structures and processes to be modified. Further, managers stated that multilateral communication within the team should be optimized with regard to treatment planning and conduct. Improving the flow of information between staff members – particularly those in different disciplines – was also important to them. In this context, managers mentioned the topics of information structures (responsibilities, clarity, and uniformity) and information control processes (via prescriptions, records, etc.) as well as the need for optimized forwarding of any information required for treatment. Managers expressed a need to ensure that staff members possessed adequate communication skills. They also perceived a need for communication training for middle management to improve discussion techniques in performance appraisal interviews. Furthermore, managers wanted to cover "new" communication media, such as email, computer-supported internal communication, and electronic patient records in the team training sessions. In addition, they wanted to establish meta-communication, meaning communication about existing communication patterns and about handling criticism.

#### Interdisciplinary team meetings

Another shared concern involves interdisciplinary team meetings (IDTMs). Managers considered IDTMs the central platform for coordinating cooperation. They were concerned with optimizing team meetings as regards (a) the course of team meetings, (b) prerequisites or framework conditions for the meetings, (c) consistent participation of all professions involved in treatment, (d) adequate forwarding of information from the meeting to the respective expert teams, (e) moderation of the meeting, (f) open communication and interaction of treatment providers, and (g) adequate documentation of the results of the team meetings. Managers outlined ideas such as how to optimize the procedure and structure of team meetings by supplying agenda items, guidelines for the meetings, specific roles, and rotating moderators. In this context, they stated that they lacked the methodological strategies to achieve the changes in the team. The rehabilitation teams also saw a need for action as regards (a) moderation of the meeting, (b) prerequisites and framework conditions for team meetings, (c) active involvement of team members in the form of multilateral communication, (d) the meeting structure, and (e) the recording and review of treatment goals. One member of the rehabilitation team stated: *"The treatment goals, to formulate them better, clearer, more informatively. To have them come across even faster. To lose less time there, or to ensure that the patient better understands what the actual goal is”* [C5_I.1_L.143-145]. The teams further saw room for improvement in the coordination of team meeting times and in the definition of time windows to ensure participation of all members. They also stated that regular meeting schedules and required participation of all professions should be ensured. In addition, it was considered necessary to train a person with social and methodological skills to play the role of mediator.

#### Team composition

Both managers and members of the rehabilitation team considered an adequate team composition as relevant to daily practice because it allows the development of a shared interdisciplinary knowledge base and of socio-emotional relationships that facilitate collaboration. They wanted to have this aspect covered in team training as well. Both sides emphasized that rather than merely bringing together the individual team members, a purposeful selection of team members was of the most importance. Regarding purposeful team composition, managers expressed the following needs: (a) tools for assessing the professional qualifications of staff members, (b) help in combining an adequate mix of skills, abilities, and personalities in the team, and (c) instruments for assessing existing teams (included professions, cultural aspects, team size, and knowledge situation). As regards the tools desired for assessing the professional qualifications of staff members, one manager asked: “*An instrument one can use, or do you know what it looks like in other areas? Do you have any experience or knowledge in this regard?*” [C2_I.1_L.286-291]. The rehabilitation team members agreed that professional qualification is a relevant aspect to be considered in team composition. In addition, they favored using assessments of so-called soft factors (motivation, work morale, and willingness to work within a team) and hard factors (practical expertise, years of professional experience) in the selection and composition of teams.

#### Coordination of treatment measures and procedures

Both groups were concerned with optimizing the coordination of treatment leadership. Managers and rehabilitation teams alike believed that a future challenge would be overcoming the traditional discipline-centered thinking in favor of interdisciplinary, team-centered thinking and action when coordinating treatment services: *"The impression that we are really a solid team, I do not have that yet. Instead, each professional group stays too separate, and keeps a certain distance to the other, different occupations"* [C3_I.10_L.128]. Both groups expressed a need for team training on (a) optimizing post-inpatient care, (b) ensuring clear assignment of tasks and responsibilities in the treatment team, and (c) defining or revising concepts for defining rehabilitation goals. Managers further expressed a need for (a) changing organizational structures and processes for optimizing treatment coordination, (b) optimizing the available time for coordinating treatment leadership, (c) decentralizing task coordination away from the departments and toward ward-based task coordination, and (d) developing a uniform biopsychosocial treatment concept. Related to the topic of interdisciplinary treatment coordination, the managers noted that existing processes in clinical inpatient settings must be revised to optimize the collaboration between physicians, therapists, nurses, and allied health professions: *"…working together on patients and therefore some of the blurred boundaries between therapeutic nursing care and physical therapy, therapeutic nursing care and speech therapy, therapeutic nursing care and occupational therapy. That is an important issue …“[C2_I.9 L.6].* The rehabilitation teams further associated team training with the desire to train a competent team coordinator who works in a goal-oriented manner, has expertise, and can work in a team.

#### Mutual appreciation

Managers and rehabilitation teams alike were concerned about mutual appreciation and respectful interactions. Both groups favored the observation of behavioral norms, such as respectful, open interaction in daily collaboration. From the managers' perspective, mutual appreciation and attitudes such as the willingness to help one another and respecting the expertise of other team members were important for interdisciplinary cooperation and should be covered in team training. They noted that this concern is not limited to interactions between managers and staff but also extends to interactions between members of the rehabilitation team. Rehabilitation team members noted that in daily collaboration, managers do not pay sufficient attention to this aspect and hoped that the training sessions would sensitize them to this topic, particularly because it can affect positively or negatively the motivation and cooperation at work. Example statements on this topic include the following: *"Problems arise because we as a department often do not talk with each other and value each other because we only see things from our own perspective, meaning occupational therapy, physical therapy, nursing, or medicine, but fail to really notice the perspectives of others. (…) well, I have always wanted for therapists (…) to go to other departments, to take other perspectives, and as a result feel much less attacked and appreciate each other more. And to me, that would be absolutely desirable"* [C2_I.4_L.596].

### Part B: Team training expectations not shared by managers and rehabilitation teams (Study Aim 2)

Figure 2 lists 13 expectations that were listed either only by managers (10 expectations) or only by rehabilitation teams (3 expectations). Team training expectations without overlap between the two groups were categorized by content into inter-individual and/or intra-individual aspects (IIA), intra-organizational aspects (IOA), and extra-organizational aspects (EOA). The first category (IIA) includes desired team training contents that primarily aim to achieve a change in attitude or in affective processes on an individual level. For instance, some listed expectations relate to changes in work morale or attitude on an individual level or between individual members of the rehabilitation team and are subject to affective changes, such as the expressed concern "optimize work morale and attitude toward teamwork." A second category relates to internal changes in organizational processes or structures that can be initiated, steered, and changed via process instructions, clinical pathways, working/project groups, or leadership behavior within the organization (IOA). For instance, some desired changes require decision-making power and are primarily the responsibility of individuals in middle or upper management, such as "improving the involvement of part-time staff" or "optimizing the formulation of treatment goals." The last category comprises desired external professional support in the form of consultation, supervision, or team analysis services (IOA). Some expressed expectations involve support for implementation from external consultants (consultation, supervision, or team analysis), for instance, "supervision regarding conflict management within the team", "consultation and supervision regarding stressful experiences in patient care" or the desired "analysis of team processes."

An analysis of differences between the expectations of managers and staff, in accordance with Study Aim 2, reveals that the latter listed expectations for team development on all levels, but particularly those affecting them personally in their daily work, namely as relates to (a) team climate, (b) conflicts within the team, and (c) high workload. The analysis reveals that managers listed various expectations in all areas affecting them personally in their roles and tasks as managers or leaders in operational or strategic management (e.g., developing a concept of interdisciplinary teamwork) and in establishing structural framework conditions (involving part-time workers), but they also fulfilled their roles as leaders and managers by listing interpersonal aspects concerning only staff members (e.g., clarity of team roles, team coherence) as expectations for team training.

## Discussion

Our exploration of team members´ and clinical managers´ expectations and concerns showed that there is a strong need for interdisciplinary training programs. Seven common core themes from the health care teams’, department heads’ and clinical managers’ perspectives emerged from the interviews, including staff participation, team and employee leadership, communication, team meetings, team composition, coordination, and mutual respect Additionally, 13 themes were identified by one of the groups but not the other (see Fig. 2). Overall, the expectations regarding team training content in healthcare require changes at the team level and organizational level. At the organizational level, a number of structural factors were mentioned (e.g., improved general conditions for team meetings, more time for making arrangements for team meetings, more flexible organization of working time, organized workshops for exchanging interdisciplinary experiences, optimization of existing communication and information structures, leadership training, instruments for the selection and transparency of qualifications, and skills of rehabilitation team members). On the team level, changes of procedural factors were considered desirable (e.g., consideration of the individual interests of all health care team members, optimization of consecutive planning and coordination of patient treatments, better knowledge integration through involvement and participation of all healthcare disciplines, improvement of multilateral communication among all team members, clarity with regard to roles and responsibilities of team members, mutual esteem and appreciation between different professions, and willingness to help each other). On the organizational level and team level, some existing studies discuss issues also identified in our study. These issues may be highly relevant for the clinical setting in medical rehabilitation. For instance, Lamb et al. 2012 [43] developed a tool in the form of a checklist (MDT QuIC) that can support the decision-making process in multidisciplinary team meetings and gives the team meeting a structural framework. For optimizing planning and coordinating patient treatment on the team level, some methods for smart goal setting and efficient goal management in rehabilitation called `Goal attainment scaling (GAS)´ are described by Bovend et al. 2009 [44] and Turner-Stokes 2009 [45], and other goal-setting methods are specified by Holliday et al. 2005 [46] and Wade 2009 [47]. To promote shared mental models (SMM), a concept that was mentioned specifically by team leaders, Gurtner et al. 2007 [48] propose the strategy of targeted reflection. Our synthesis underscores that there is a meaningful heterogeneity of team-training needs. Therefore, training interventions need to be highly customized to meet the requests of both executives and team members at the rehabilitation clinic. Caroll 2006 [49] points out that team training interventions must be tailored to fit the dynamics of each team and the needs of an organization. Also, Baker, Day & Salas 2006 [50] draw attention to the importance of adapting team training to specific health care needs of an organization because health care services operate in a number of diverse contexts with diverse needs (e.g., emergency medicine and rehabilitation medicine). In addition, the narrative review by Weaver et al. 2014 [13] presents five fixed team training strategies: (a) assertiveness training, (b) cross-training, (c) error management training,(d) metacognition training, and (e) team adaptation and coordination training. In accordance with the findings of Weaver et al. 2014 [13], some individual elements of training content such as conflict management, mutual trust, team leadership, communication competence, and shared mental models of team roles and responsibilities were also formulated in our investigation as concerns for team training interventions.

In summary, we did not find previous studies that investigated the expectations of rehabilitation team members or clinical managers concerning training content in health care before conceptualizing a training program. However, modularized team training programs exist whose contents were developed as structured modules based on the literature and that were empirically tested after the practical implementation of the program [13]. In this context, respondents were asked about the relevance of the training content after completing the intervention. In our approach, in contrast, participants were interviewed in advance in order to determine the need for team training. In line with the results of the “modularized Crew Resource Management Training for Health Care Professionals” by Clay-Williams et al. 2014 [51], the participants surveyed regarded the following topics, which are similar to those found in our study, as substantially relevant training content: (a) to learn tools and strategies to improve general communication and team skills, (b) to optimize interdisciplinary communication, (c) to develop shared mental models, (d) to improve quality in health care, (e) to share experiences with others, (f) to learn about conflict resolution, (g) to learn something new or interesting, and (h) to improve organization or time management. Other identified aspects that were not mentioned in our study are (a) to learn about assertiveness, (b) to develop briefing strategies, and (c) to improve situational awareness. However, there are different approaches to individualizing team-training programs by taking into account the specific conditions within an organization. Most training programs that can be found in the literature are modularized programs that are not adapted specifically to the needs of an organization. However, the high heterogeneity of demands on the content of team training that we found in our study calls for a more individualized training approach. Clay-Williams et al. 2014 [51] argue in favor of modularized training programs. They point out that modular training allows health professionals to choose topics individually that are relevant to their workplace and to add other topics when needed. However, we would like to point out that such intervention programs still may not be flexible enough to adapt to the current conditions of the organizations. We believe that only tailored team interventions can meet the needs of the complex environment of rehabilitation clinics. Overall, our study results and the results of the narrative review of team interventions in chronic disease rehabilitation by Körner et al. 2014 [52] indicate a lack of training programs for staff in medical rehabilitation clinics in Germany.

### Strengths and limitations

The data were collected in 18 semi-structured interviews and 5 focus groups. This qualitative methodology allowed us to gather novel insights with a degree of depth that was not achievable from surveys. It is worth noting that the application of different data collection methods may affect the results of the study. The idea to carry out interviews with the experts instead of a focus group resulted from the assumption that clinic managers are in competition with one another because they belong to different clinic groups or groups of companies. If a common focus group were conducted with all managers of rehabilitation clinics in the same field, it is conceivable that respondents would not reply as openly and answers would be more superficial. We considered individual interviews as useful for gathering data on sensitive issues. Therefore, we preferred conducting face-to-face interviews with clinical managers and focus groups with staff members. To standardize the process, however, an interview guide with roughly the same items for individual interviews and for focus groups was used to ensure that important areas were discussed and a standardized opening question was used at the beginning of the interview. All interviews were performed by two researchers who intended to keep the interview situation comparable between participants. The fact that the analysis was based on continuous discussion between the authors throughout the process increases the reliability of the analysis. Moreover, the credibility of the results was validated by each of the two researchers (CM and AP) reading and analyzing them separately before comparing and confirming the arising categories. However, the current study has some limitations. This study gathered information about expectations regarding team-training interventions in rehabilitation settings from 53 participants at five clinics in Germany. Our findings cannot simply be transferred directly between countries without taking into account differences in professional culture, professional education, or clinical practice, and they may not be applicable to other settings, other fields, and other populations of health professionals. Furthermore, it is necessary to consider the social, political, and geographical context of the findings because of the diverse healthcare systems in other countries. In terms of the recruitment procedure, it is likely that a selection bias operated in participants who agreed to take part in the research and who were selected by a clinical manager. They may have been more interested in team training programs than those individuals who chose not to participate or were not asked to participate. Therefore, a selection bias cannot be excluded. It should further be critically noted that the inclusion of guiding questions for the interview narrowed the range of responses interviewees may give. Also the interests of respondents in leadership positions (e.g., clinical managers or heads of department), who primarily represent the clinics’ interests, could have distorted the results. Moreover, interviewer experience and knowledge as well as the composition of the focus groups and participants’ mutual perception and impressions of each other in the interview situation (e.g., mutual like or dislike) could have influenced the response behavior and answers of the respondents.

### Implications for practice

The results have several practical implications for team training. Our study introduced seven main categories of the greatest demands and needs for team training content as expressed by health care team members and clinical managers. Thus, managers and administrators of medical rehabilitation clinics should consider addressing those needs of health professionals working in their clinics in order to improve the quality of care. Tools such as staff appraisal interviews, questionnaires, or organizational performance standards that particularly include this dimension can help to determine the individual training needs. Health care managers and administrators need to develop interpersonal skills in order to successfully run complex organizations and must be able to coach others. Therefore, leadership coaching has been an underutilized resource in health care executive training, and establishing training programs on these topics should be considered. The diversity of topics brought up in the interviews shows that it may be difficult to adapt standardized team training programs to the complex environments of specific clinics or to transfer existing modularized training programs from acute-care settings in the context of the German health care system. Therefore, we are in favor of training programs that are standardized regarding didactic processes and methodologies but not regarding specific session content because individual organizations may differ widely in terms of content needs. Team training should also be integrated in a comprehensive educational approach and organizational context with the goal of improving cooperation and coordination between and among health professionals´, rather than simply forming better teams or performing team training when problems arise. For this reason, interdisciplinary teamwork training should be introduced early in healthcare training, for example when starting work at the rehabilitation clinic, or it should be conducted regularly or periodically in order to promote team development in health care.

## Conclusions

To our knowledge, this is the first qualitative study relevant for researchers and practitioners alike that provides insight into health care team members´ and clinical managers´ expectations concerning team training content. By drawing upon the study results and discussing existing team training concepts, we have provided a preliminary proposal for developing a team training approach for the medical rehabilitation setting in Germany: training interventions should be based on the specific needs and concerns of the individual rehabilitation clinic and should include a systemic, goal-oriented, task-oriented, and solution-oriented strategy. We regard this strategy as a feasible delivery method for teamwork training. First, it is practical and applicable in different specializations. Second, it promotes interdisciplinary and collaborative relationships within and between disciplines. And third, it closely reflects the individual needs orientation of rehabilitation clinics and is tailored to their environments. We believe that non-modular training may open the door to further innovations in team training research initiatives. Our hope is that this effort not only highlights the need for developing new team training interventions to improve team performance but will also initiate discussion on the need of team training in general. It is becoming quite clear that some of the biggest challenges to be solved are finding the right fit between team needs and organizational needs as well as determining the intervention content, the training modalities, and the strategies for implementing team training in daily practice. Future work should evaluate team-training efforts across time, implementation strategies, and learning outcomes in medical rehabilitation in Germany. This includes examining the impact on patient satisfaction and team satisfaction outcomes when using robust training strategies and implementation designs as well as evaluating team-training effects.
